# Paternal and maternal obesity but not gestational weight gain is associated with type 1 diabetes

**DOI:** 10.1093/ije/dyx266

**Published:** 2018-02-05

**Authors:** Maria C Magnus, Sjurdur F Olsen, Charlotta Granstrom, Nicolai A Lund-Blix, Jannet Svensson, Jesper Johannesen, Abigail Fraser, Torild Skrivarhaug, Geir Joner, Pål R Njølstad, Ketil Størdal, Lars C Stene

**Affiliations:** 1 Division for Mental and Physical Health, Norwegian Institute of Public Health, Oslo, Norway; 2 MRC Integrative Epidemiology Unit at University of Bristol, Bristol, UK; 3 Department of Population Health Sciences, Bristol Medical School, Bristol, UK; 4 Centre for Fetal Programming, Department of Epidemiology Research, Statens Serum Institut, Copenhagen, Denmark; 5 Department of Pediatrics, Oslo University Hospital, Oslo, Norway; 6 Department of Pediatrics, Copenhagen University Hospital, Herlev, Denmark; 7 NIHR Bristol Biomedical Research Centre at the University Hospitals Bristol, NHS Foundation Trust and the University of Bristol, Bristol, UK; 8 Institute of Clinical Medicine, University of Oslo, Oslo, Norway; 9 Department of Pediatrics, Haukeland University Hospital, Bergen, Norway; 10 KG Jebsen Center for Diabetes Research, Department of Clinical Science, University of Bergen, Bergen, Norway; 11 Department of Pediatrics, Ostfold Hospital Trust, Fredrikstad, Norway

**Keywords:** body mass index, parents, pregnancy, type 1 diabetes, weight gain

## Abstract

**Background:**

Our objective was to examine the associations of parental body mass index (BMI) and maternal gestational weight gain with childhood-onset type 1 diabetes. Comparing the associations of maternal and paternal BMI with type 1 diabetes in the offspring will provide further insight into the role of unmeasured confounding by characteristics linked to BMI in both parents.

**Methods:**

We studied 132 331 children participating in the Norwegian Mother and Child Cohort Study (MoBa) and the Danish National Birth Cohort (DNBC) who were born between February 1998 and July 2009. Exposures of interest included parental BMI and maternal gestational weight gain obtained by maternal report. We used Cox-proportional hazards regression to examine the risk of type 1 diabetes (*n*=499 cases), which was ascertained by national childhood diabetes registers.

**Results:**

The incidence of type 1 diabetes was 32.7 per 100 000 person-years in MoBa and 28.5 per 100 000 person-years in DNBC. Both maternal pre-pregnancy obesity, adjusted hazard ratio (HR) 1.41 [95% confidence interval (CI): 1.06, 1.89] and paternal obesity, adjusted HR 1.51 (95% CI: 1.11, 2.04), were associated with childhood-onset type 1 diabetes. The associations were similar after mutual adjustment. In contrast, maternal total gestational weight gain was not associated with childhood-onset type 1 diabetes, adjusted HR 1.00 (95% CI: 0.99, 1.02) per kilogram increase.

**Conclusions:**

Our study suggests that the association between maternal obesity and childhood-onset type 1 diabetes is not likely explained by intrauterine mechanisms, but possibly rather by unknown environmental factors influencing BMI in the family.

**Key Messages**

- We observed similar positive associations of both maternal and paternal obesity with childhood-onset type 1 diabetes in two large pregnancy cohorts.
- Our study therefore suggests that the previously reported association between maternal obesity and childhood-onset type 1 diabetes is not explained by intrauterine mechanisms, but possibly rather by unknown environmental factors influencing body mass index in the family.

## Background

Evidence suggests that the early-life environment influences the risk of type 1 diabetes.^1^^,^^2^ For example, the risk of type 1 diabetes is inversely associated with birth weight,^3^^,^^4^ potentially because of the effect of nutrient transfer *in utero* on fetal development of the pancreas.^2^ The risk of type 1 diabetes also increases with infant weight gain, further suggesting a role for postnatal nutritional status.^5–8^ Maternal pre-pregnancy obesity and excessive gestational weight gain are proposed to exert a broad influence on fetal development, as a result of insulin resistance leading to hyperinsulinemia, inflammation and oxidative stress, which may all contribute to placental dysfunction.^9^

The previous studies that examined the association between maternal pre-pregnancy body mass index (BMI) and risk of either type 1 diabetes or autoimmunity in offspring reported both no association^10–12^ and a positive association.^13^^–^^16^ Likewise, studies of maternal gestational weight gain in relation to offspring type 1 diabetes reported mixed findings, with studies reporting both no association^10^^,^^16^^,^^17^ and a positive association.^15^^,^^18^^,^^19^ The discrepancies may be due to different study designs, outcome definitions (type 1 diabetes vs islet autoimmunity), statistical power to detect associations or the fact that several previous studies were restricted to individuals of genetically high risk.

One way to explore the likelihood of unmeasured confounding in observational studies is to use negative controls.^20^^,^^21^ Paternal BMI can be used as a negative control for an *in utero* effect of maternal pre-pregnancy BMI on offspring outcomes. If similar associations are observed with maternal and paternal BMI, this suggests that the associations with offspring outcomes, here type 1 diabetes, most likely reflect a potential influence of genetic or environmental characteristics linked to BMI in both parents. Paternal BMI has not previously been examined in relation to type 1 diabetes.

The objective of our study was therefore to examine the associations of parental BMI and maternal gestational weight gain with childhood-onset type 1 diabetes.

## Methods

### Study population

**Figure 1:**
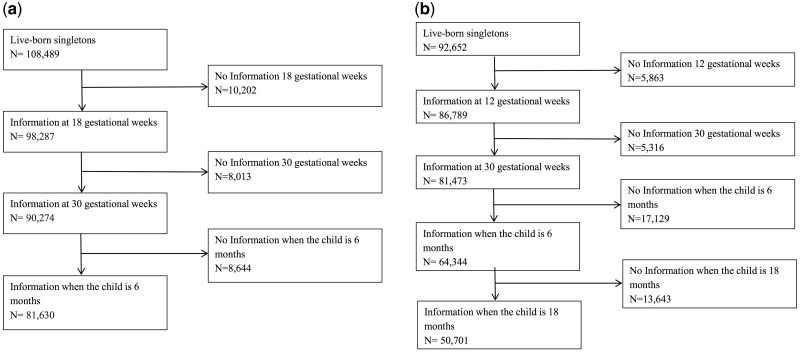
Formation of the analysis sample. (a) The Norwegian Mother and Child Cohort Study (MoBa). (b) The Danish National Birth Cohort (DNBC). In MoBa, a total of 2545 children were excluded because they were stillborn, abortions or had unknown birth outcome, and 3842 live-born children were excluded because they were from multiple births. In DNBC, a total of 6303 children were excluded because they were stillborn, abortions or had unknown birth outcome, and 4163 live-born children were excluded because they were from multiple births.

The Norwegian Mother and Child Cohort Study (MoBa) is a population-based pregnancy cohort administered by the Norwegian Institute of Public Health.^22–24^ MoBa recruited pregnant women across Norway between 1999 and 2008, at approximately 18 gestational weeks. Of the eligible women, 41% participated, and all participants gave a written informed consent. We used data available in March 2014 (version VIII of the quality assured data files), comprising 95 267 mothers with 114 761 live-born children. A total of 81 630 singletons in MoBa had information from questionnaires administered at 18 gestational weeks, 30 gestational weeks and when the child was 6 months, and were subsequently included in the current study (Figure 1a). The Norwegian Data Inspectorate and the Regional Ethics Committee for Medical Research of South East Norway approved this study.

The Danish National Birth Cohort (DNBC) is a population-based pregnancy cohort administered by the Statens Serum Institut in Denmark.^25^^,^^26^ Pregnant women were recruited at their first antenatal visit across Denmark between 1996 and 2002. Approximately 50% of all general practitioners in Denmark participated in the recruitment, and 60% of invited women agreed to participate. The cohort consists of 91 745 mothers and 103 118 children. All participants gave a written informed consent. A total of 50 701 singletons from DNBC with information gathered through telephone interviews at 12 and 30 gestational weeks, in addition to information collected through interviews when the child was 6 and 18 months old, were included in the current study (Figure 1b). The data collection in the DNBC is approved by the Danish National Ethics Board.

### Parental height, weight and gestational weight gain

Information regarding maternal weight at the beginning of pregnancy, maternal weight at the end of pregnancy, maternal height, paternal height and paternal weight were obtained by maternal report through self-completed questionnaires (MoBa) and interviews (DNBC). Notably, paternal height and weight were obtained at recruitment at 18 gestational weeks in MoBa, and when the child was 18 months in DNBC. The main exposures included parental BMI (weight in kg/height in m^2^) and maternal gestational weight gain throughout pregnancy (in kg). Parental BMI was evaluated both continuously and categorized according to underweight (<18.5), normal weight (18.5–24.9), overweight (25–29.9) and obese (30 and higher).

### Childhood-onset type 1 diabetes

The outcome was a clinical diagnosis of type 1 diabetes, ascertained by the Norwegian Childhood Diabetes Register and the Danish Childhood Diabetes Register—both nationwide registers with a high level of case ascertainment.^27^^,^^28^ Information on new-onset doctor-diagnosed type 1 diabetes is reported to the registers from all the pediatric departments in Norway and Denmark. Cases of type 1 diabetes are therefore defined based on a clinical diagnosis, using the first insulin injection as the date of diagnosis. Rare cases of type 2 diabetes or monogenic diabetes were excluded from both registers.

### Covariates

We obtained information on a number of characteristics that might plausibly be related to both parental BMI, maternal gestational weight gain and childhood-onset type 1 diabetes. Parental age at the time of delivery, maternal parity, the child’s gender and birthweight were obtained from national birth registers. In addition, parental education and parental smoking during pregnancy were obtained by self-report in both cohorts. Finally, information on both maternal and paternal history of type 1 diabetes was obtained through the Norwegian Patient Registry for MoBa participants, whereas maternal history of all types of diabetes was gathered through a national diabetes registry for DNBC participants.^29^ Paternal history of diabetes was therefore not available for DNBC participants.

### Statistical analysis

We examined the associations of parental BMI and maternal gestational weight gain with childhood-onset type 1 diabetes using Cox-proportional hazards regression separately for each cohort, reporting hazards ratios (HRs) and 95% confidence intervals (CIs). We subsequently combined the results from the two cohorts by a random-effects meta-analysis. Heterogeneity of the associations observed in the two cohorts was examined by the I^2^ statistic. Participants were followed from their date of birth until diagnosed as having type 1 diabetes or the end of follow-up (21 July 2016 for MoBa and 15 May 2016 for DNBC). A robust cluster variance estimation was used to account for the presence of siblings. We evaluated the proportional hazards assumption by examining the Schoenfeld residuals. The multivariable analysis of maternal pre-pregnancy BMI in relation to childhood-onset type 1 diabetes adjusted for maternal age, parity, education, smoking during pregnancy and history of diabetes. The analysis of gestational weight gain further adjusted for the child’s gender, in addition to these maternal background characteristics. The multivariable analysis of paternal BMI adjusted for paternal age, education, smoking during pregnancy and type 1 diabetes (MoBa only).

We explored whether there was any nonlinear associations between maternal gestational weight gain and childhood-onset type 1 diabetes using a likelihood ratio tests to compare the models with and without a second-order term. The direct association between maternal pre-pregnancy BMI and childhood type 1 diabetes not mediated by birthweight and infant weight gain the first year of life was estimated by including these two covariates in the multivariable model. We have previously reported a positive association between infant weight gain and childhood-onset type 1 diabetes in these two pregnancy cohorts, whereas there was no strong evidence of an association with birthweight [8]. For the MoBa cohort, we also further evaluated potential mediation by BMI at 36 months in the sub-sample with follow-up information from the 36-month questionnaire (*n*=53 571). To account for missing covariate information within the defined study sample (*n*=81 630 from MoBa; *n*=50 701 from DNBC), we conducted multiple imputation of missing data using chained equations, generating 20 imputed datasets.

The analysis was conducted using SAS version 9.4 (SAS Institute, North Carolina, USA) and Stata version 14 (StataCorp, Texas, USA).

## Results

A total of 132 331 children were available for analysis (Figure 1). The children included in the study were born between February 1998 and July 2009. Maternal education tended to be higher, smoking less common and parity lower among mothers of children included in the analysis compared with those excluded because of insufficient follow-up information (Supplementary Table 1, available as Supplementary Data at *IJE* online). Notably, the risk of type 1 diabetes was similar among those not responding to follow-up questionnaires/interviews and those who did (Supplementary Figures 1 and 2, available as Supplementary Data at *IJE* online). The mean age of the children at the end of follow-up was 11.0 years (range 7.0, 17.5 years) in MoBa and 15.5 years (range 13.0, 18.2 years) in DNBC. The incidence rate of type 1 diabetes was 32.7 per 100 000 person-years in MoBa and 28.5 per 100 000 person-years in DNBC. Overall, the background characteristics in the two cohorts were very similar (Table 1).

**Table 1.** Distribution of background characteristics

Characteristic	MoBa (*n* = 81 630)	DNBC (*n* = 50 701)
**Maternal age [mean (SD)]**	30.2 (4.5)	30.6 (4.2)
**Maternal parity [*n* (%)]**		
Primiparous	37 413 (45.8)	23 061 (45.5)
1	28 731 (35.2)	19 004 (37.5)
2+	15 486 (19.0)	8621 (17.0)
Missing	0 (0.0)	15 (0.03)
**Maternal education [*n* (%)]**		
Less than high school (MoBa)/9^th^ grade with exam (DNBC)	5579 (6.8)	3672 (7.2)
High school (MoBa)/10^th^ grade with exam (DNBC)	23 452 (28.7)	13 687 (27.0)
Up to 4 years of college (MoBa)/Technical school (DNBC)	33 680 (41.3)	6886 (13.6)
More than 4 years of college (MoBa)/ High school or more (DNBC)	18 576 (22.8)	25 969 (51.2)
Missing	343 (0.4)	507 (1.0)
**Maternal smoking during pregnancy [*n* (%)]**		
No	62 963 (77.1)	37 954 (74.9)
Yes	18 346 (22.5)	12 746 (25.1)
Missing	321 (0.4)	1 (0.0)
**Maternal diabetes [*n* (%)]^a^**		
No	81 268 (99.6)	49 316 (97.3)
Yes	362 (0.4)	1385 (2.7)
**Maternal pre-pregnancy body mass index [*n* (%)]**		
Underweight	2363 (2.9)	2066 (4.1)
Normal weight	52 433 (64.2)	33 614 (66.3)
Overweight	17 474 (21.4)	9996 (19.7)
Obese	7375 (9.0)	4211 (8.3)
Missing	1985 (2.4)	814 (1.6)
**Maternal gestational weight gain [mean (SD)]**	14.9 (5.8)	14.9 (5.9)
Missing [*n* (%)]	6096 (7.5)	529 (1.0)
**Paternal age [*n* (%)]**		
Less than 25	3566 (4.4)	1656 (3.3)
25–29	18 446 (22.6)	13 184 (26.0)
30–34	31 934 (39.1)	20 011 (39.5)
35+	27 492 (33.7)	15 232 (30.0)
Missing	192 (0.2)	618 (1.2)
**Paternal education [*n* (%)]**		
Less than high school (MoBa)/9^th^ grade with exam (DNBC)	8026 (9.8)	9430 (18.6)
High school (MoBa)/10^th^ grade with exam (DNBC)	31 845 (39.0)	17 608 (34.7)
Up to 4 years of college (MoBa)/Technical school (DNBC)	21 728 (26.6)	2784 (5.5)
More than 4 years of college (MoBa)/ High school or more (DNBC)	17 609 (21.6)	18 928 (37.3)
Missing	2422 (3.0)	1951 (3.8)
**Paternal smoking (MoBa)/Partner smoking (DNBC) [*n* (%)]**		
No	59 930 (73.4)	35 827 (70.7)
Yes	21 301 (26.1)	14 834 (29.3)
Missing	399 (0.5)	40 (0.1)
**Paternal type 1 diabetes [*n* (%)]**		
No	81 106 (99.4)	NA
Yes	524 (0.6)	NA
**Paternal pre-pregnancy body mass index [*n* (%)]**		
Underweight	163 (0.2)	191 (0.4)
Normal weight	34 971 (42.8)	25 499 (50.3)
Overweight	35 313 (43.3)	19 274 (38.0)
Obese	7544 (9.2)	3475 (6.8)
Missing	3639 (4.5)	2262 (4.5)
**Child gender [*n* (%)]**		
Male	41 796 (51.2)	25 773 (50.8)
Female	39 834 (48.8)	24 928 (49.2)
**Child birth weight [mean (SD)]**	3.6 (0.5)	3.6 (0.5)
Missing	99 (0.1)	251 (0.5)
**Child weight gain the first year of life in kg [mean (SD)]**	6.3 (1.0)	6.6 (1.1)
Missing	21 430 (26.3)	9403 (18.5)
**Child type 1 diabetes [*n* (%)]**		
No	81 337 (99.6)	50 495 (99.6)
Yes	293 (0.4)	206 (0.4)
**Child age at type 1 diabetes diagnosis [median (range)]**	7.1 (0.7, 15.0)	9.6 (0.9, 17.0)

a Maternal diabetes includes only type 1 diabetes in MoBa whereas it includes all forms of diabetes in DNBC.

### Parental BMI in relation to the offspring’s risk of type 1 diabetes

Maternal pre-pregnancy obesity (pre-pregnancy BMI ≥30) was associated with an increased risk of childhood-onset type 1 diabetes, with a combined HR of 1.41 (95% CI: 1.06, 1.89) compared with offspring of mothers with a normal pre-pregnancy BMI (Table 2). Interestingly, we observed a very similar association between paternal obesity and risk of childhood-onset type 1 diabetes, with a combined a HR of 1.51 (95% CI: 1.11, 2.04) as compared with children of normal-weight fathers (Table 2). The magnitude of the association between parental BMI and childhood-onset type 1 diabetes tended to be greater MoBa than in DNBC, but we only observed strong evidence of heterogeneity in the association between maternal overweight and risk of type 1 diabetes (Table 2). The results from the complete-case analyses yielded similar associations (Supplementary Table 2, available as Supplementary Data at *IJE* online).

**Table 2.** Associations of maternal pre-pregnancy body mass index and paternal body mass index with the risk of type 1 diabetes

Parent	Study	Exposure	Person-years	Events	Unadjusted HR (95% CI)	Adjusted HR (95% CI)	Test of heterogeneity (*p*-value)
Mother	MoBa	Continuous	896 679	293	1.05 (1.03, 1.08)	1.05 (1.02, 1.07)	
		Underweight	26 400	9	1.31 (0.67, 2.56)	1.33 (0.68, 2.59)	
		Normal weight	588 534	158	1	1	
		Overweight	197 983	88	1.64 (1.25, 2.15)	1.60 (1.21, 2.10)	
		Obese	83 762	38	1.67 (1.17, 2.40)	1.57 (1.09, 2.27)	
	DNBC	Continuous	722 194	206	1.01 (0.97, 1.04)	1.00 (0.96, 1.03)	
		Underweight	37 816	11	1.28 (0.69, 2.37)	1.30 (0.70, 2.40)	
		Normal weight	472 585	138	1	1	
		Overweight	127 823	33	0.79 (0.54, 1.17)	0.75 (0.51, 1.11)	
		Obese	83 970	24	1.41 (0.91, 2.18)	1.18 (0.73, 1.88)	
	Combined	Continuous	1 618 873	499	1.03 (0.99, 1.07)	1.03 (0.98, 1.08)	0.025
		Underweight	64 216	20	1.29 (0.82, 2.04)	1.31 (0.84, 2.07)	0.961
		Normal weight	1 061 119	296	1	1	NA
		Overweight	325 806	121	1.15 (0.56, 2.36)	1.11 (0.53, 2.33)	0.002
		Obese	167 732	62	1.56 (1.18, 2.06)	1.41 (1.06, 1.89)	0.350
Father	MoBa	Continuous	896 679	293	1.06 (1.02, 1.10)	1.05 (1.01, 1.09)	
		Underweight	1868	1	1.97 (0.27, 14.10)	1.89 (0.26, 13.75)	
		Normal weight	402 983	113	1	1	
		Overweight	405 868	136	1.19 (0.92, 1.54)	1.17 (0.90, 1.51)	
		Obese	85 961	43	1.78 (1.24, 2.57)	1.67 (1.15, 2.43)	
	DNBC	Continuous	722 194	206	1.01 (0.97, 1.06)	1.01 (0.97, 1.06)	
		Underweight	3824	1	1.22 (0.17, 8.73)	1.21 (0.17, 8.63)	
		Normal weight	351 465	104	1	1	
		Overweight	304 472	82	1.04 (0.77, 1.40)	1.06 (0.79, 1.43)	
		Obese	62 433	19	1.34 (0.81, 2.21)	1.40 (0.85, 2.32)	
	Combined	Continuous	1 618 873	499	1.04 (0.99, 1.09)	1.03 (0.99, 1.07)	0.19
		Underweight	5692	2	1.55 (0.38, 6.25)	1.51 (0.37, 6.09)	0.75
		Normal weight	754 448	217	1	1	NA
		Overweight	710 340	218	1.12 (0.92, 1.37)	1.12 (0.92, 1.36)	0.62
		Obese	148 394	62	1.61 (1.20, 2.17)	1.51 (1.11, 2.04)	0.72

Maternal pre-pregnancy body mass index adjusted for maternal age, parity, education, smoking status during pregnancy and diabetes (type 1 in MoBa and all types in DNBC). Paternal body mass index adjusted for paternal age, education, smoking (paternal smoking in MoBa and maternal partner smoking in DNBC) and type 1 diabetes (MoBa only). Multiple imputation of missing exposure and covariate information using chained equations. A total of 20 datasets were imputed.

The association observed between maternal obesity and childhood-onset type 1 diabetes remained after including birth weight and infant weight gain in the first 12 months of life in the multivariable model (Supplementary Table 3, available as Supplementary Data at *IJE* online). In the sub-sample of MoBa participants with information from the 36-month questionnaire, further adjustment for BMI at 36 months did not change the association between maternal BMI and childhood-onset type 1 diabetes (data not shown). Since maternal and paternal BMIs show a modest correlation (Pearson correlation coefficient of 0.23 in MoBa and 0.18 in DNBC), we conducted an analysis where these two exposures were mutually adjusted for each other. This analysis indicated that the associations observed between maternal and paternal obesity in relation to offspring type 1 diabetes were independent (Supplementary Table 4, available as Supplementary Data at *IJE* online).

### Maternal gestational weight gain and the offspring’s risk of type 1 diabetes

There was no associations between maternal total gestational weight gain throughout pregnancy and risk of childhood-onset type 1 diabetes (Table 3). The association was similar in the complete-case analysis (Supplementary Table 5, available as Supplementary Data at *IJE* online). Including a second-degree term indicated no strong evidence of any nonlinear associations (*p*-value 0.36 in MoBa and 0.07 in DNBC).

**Table 3.** Association between maternal total gestational weight gain and the risk of type 1 diabetes

Study	Mean (SD)	Person-years	Events	Unadjusted HR (95% CI)	Adjusted HR (95% CI)	Test of heterogeneity (*p*-value)
MoBa	14.9 (5.8)	896 679	293	1.00 (0.98, 1.01)	1.00 (0.98, 1.02)	
DNBC	14.9 (5.9)	722 194	206	1.00 (0.97, 1.03)	1.00 (0.98, 1.03)	
Combined	14.9	1 618 873	499	1.00 (0.98, 1.02)	1.00 (0.99, 1.02)	1.00

Adjusted for maternal age, parity, education, smoking status during pregnancy, diabetes (type 1 in MoBa and all types in DNBC), weight at the start of pregnancy and child gender. Multiple imputation of missing exposure and covariate information using chained equations. A total of 20 datasets were imputed. Including a second-degree term indicated no evidence of any nonlinear associations (*p*-value 0.36 in MoBa and 0.07 in DNBC).

## Discussion

Our study including two of the world’s largest pregnancy cohorts revealed that maternal obesity before pregnancy, but not gestational weight gain, is associated with an increased risk of developing childhood-onset type 1 diabetes. Interestingly, we also found that paternal obesity showed a similar association with type 1 diabetes in the offspring. To our knowledge, this is the first report on the association between paternal obesity and childhood-onset type 1 diabetes. As we discuss below, our interpretation of these findings is that maternal obesity is unlikely to influence type 1 diabetes risk via intrauterine nutritional mechanisms.

### Comparison with previous studies

Our findings are in line with previous studies reporting a positive association between maternal pre-pregnancy BMI and offspring type 1 diabetes or autoimmunity.^13–16^ Two population-based studies indicated a greater risk of type 1 diabetes among children of obese mothers compared to children of normal weight mothers, with an incidence rate ratio (IRR) of 1.25 (95% CI: 1.13, 1.38)^14^ and an OR 1.18 (95% CI: 1.02, 1.36),^16^ respectively. An American population-based case–control study also provided evidence that maternal obesity before pregnancy is associated with increased risk of type 1 diabetes in the offspring, with an OR of 1.29 (95% CI: 1.01, 1.64).^13^ In the Norwegian MIDIA study, which included only individuals with the highest risk of type 1 diabetes based on their human leukocyte antigen (HLA) genotype, maternal BMI was positively associated with islet autoimmunity,^15^ but this was not supported by a similar Finnish study.^10^

The results from our study do not support previous findings suggesting a greater risk of type 1 diabetes among children of mothers with a higher gestational weight gain.^15^^,^^18^^,^^19^ A British population-based cohort of 196 age- and sex-matched sets indicated that maternal excessive weight gain during pregnancy was associated with higher risk of type 1 diabetes, with an OR of 7.12 (95% CI: 1.50, 33.79).^18^ Another population-based study of 68 diabetic children and 68 sibling controls from Belgrade also reported a higher proportion of maternal gestational weight gain of 15 kg or more among the cases of type 1 diabetes than the controls (48.5% among the cases and 32.4% among the controls, *p*-value: 0.056).^19^ Finally, the MIDIA study reported a positive association with maternal gestational weight gain with islet autoimmunity.^15^

### Interpretation and potential explanatory mechanisms

A priori, we hypothesized that high maternal BMI during pregnancy could result in a nutrient overload in the offspring with implications for the developing beta-cells. Birth weight and infant weight gain have previously been associated with type 1 diabetes,^3^^–^^8^ but interestingly did not explain much of the observed association between maternal pre-pregnancy obesity and type 1 diabetes. In MoBa, further adjustment for offspring BMI at 36 months also did not change our findings. However, we cannot rule out the possibility that later childhood obesity, not captured by our adjustment, could explain our observed associations between parental obesity and childhood-onset type 1 diabetes.^30^ It may be that common family environmental characteristics conducive of obesity exerts a stronger influence on later childhood obesity.^31^

Based on the similar associations of maternal and paternal obesity with childhood-onset type 1 diabetes, it seems likely that the previously observed association between maternal obesity and type 1 diabetes reflects unmeasured genetic and/or lifestyle characteristics linked to obesity in both parents.^20^^,^^21^ Obvious lifestyle characteristics linked to obesity include diet and physical activity. Some aspects of maternal diet during pregnancy and infant diet are proposed to play a role in development of type 1 diabetes.^32^^,^^33^ For example, maternal vitamin D and n-3 fatty acid levels during pregnancy are inversely associated with type 1 diabetes, but their causal role remains speculative.^33^ Both of these nutrients are also inversely associated with BMI.

There is some evidence that HLA genotypes that increase the risk of type 1 diabetes might influence obesity, but the results are inconclusive.^34^^,^^35^ Furthermore, the BMI-associated variant near the FTO locus (in the child) is not associated with type 1 diabetes.^36^ Maternal pre-pregnancy BMI also influences DNA methylation in the offspring as measured in cord blood, but none of the current findings of differential methylation linked to maternal BMI has a particular relevance for type 1 diabetes.^37–39^ Based on the current knowledge, it is therefore unlikely that genetic or epigenetic mechanisms explain our findings, but the possibility cannot be fully excluded.

If there is a direct intrauterine programming of maternal obesity on the child’s risk of type 1 diabetes, this could be explained by the chronic low-grade inflammation that is observed among obese individuals,^40^ as this might theoretically exert an influence on the developing immune system of the fetus.^41^ For this potential mechanism to underlie the observed associations of both maternal and paternal obesity with type 1 diabetes, any influence of inflammation on paternal sperm quality would have to also impact disease risk in the offspring.

### Strengths and limitations

This study has several important strengths, including its size, the prospective design, the inclusion of two relatively homogeneous populations and the ascertainment of type 1 diabetes from national childhood diabetes registers. A few limitations should also be acknowledged.

Selection bias might have resulted from the modest participation rates and/or loss-to-follow-up, but a comparison of a number of associations in national registries indicated that the characteristics of the initial participants in the cohorts might not be a strong source of bias.^23^^,^^25^ Relying on self-report to obtain information on parental height and weight could have resulted in misclassification. If we assume that most individuals are likely to under-report rather than over-report their weight, such a misclassification could have attenuated our findings, but we cannot exclude a potential differential misclassification by other lifestyle characteristics. The mothers of children included in our analysis seemed to include a more health-conscious group (higher educational level and lower proportion of smokers), who are likely to have a lower pre-pregnancy BMI and gestational weight gain. Any such selection factors by maternal background characteristics would also have to confer a differential risk of type 1 diabetes to introduce a selection bias.^42^ Since the incidence of type 1 diabetes in the two cohorts was similar among children with and without sufficient follow-up information, indicating that the selection factors have not resulted in a differential risk of the outcome, we believe that the direction of any selection bias is more likely to be towards than away from the null. These potential selection factors would have to be more strongly associated with gestational weight gain than parental BMI to explain the fact that we observed associations with one and not the other. The association between paternal obesity and childhood-onset type 1 diabetes also tended to be of a greater magnitude in MoBa than in DNBC. Overall, the characteristics of the two cohorts are similar. If the associations between parental obesity and type 1 diabetes is non-causal, and more likely to be due to unmeasured background characteristics linked to obesity in both parents, then any heterogeneity in the findings might be explained by differences in such background characteristics.

We also relied on maternal report of paternal height and weight in both cohorts. However, paternal measurements were available also by paternal report for a subgroup of MoBa participants (approximately 70%), which showed correlation coefficients greater than 0.95 between maternal and paternal report. For DNBC, information on paternal BMI was obtained when the child was 18 months. Due to the tracking of BMI in adulthood,^43^ this measure should be representative of the father’s BMI before/during pregnancy. However, we speculate that paternal weight might have slightly increased over this time, which could have led to overestimation of paternal BMI. Unfortunately, we did not have any additional information available to further explore the validity of this measure. We also did not have information available on paternal diabetes in DNBC. This might have led us to overestimate the association between paternal BMI and childhood-onset type 1 diabetes in this cohort. However, paternal type 1 diabetes is too rare to explain much of the observed association, and is likely only weakly associated with paternal BMI. Finally, adjusting the association for paternal type 1 diabetes in MoBa did not attenuate the association. We therefore think that it is unlikely that the association observed in DNBC is biased by lack of adjustment for paternal type 1 diabetes.

## Conclusion

This study of two of the world’s largest pregnancy cohorts revealed a positive association between both maternal and paternal obesity and childhood-onset type 1 diabetes. Our study suggest that the association between maternal obesity and childhood-onset type 1 diabetes observed in our and other studies is likely explained not by intrauterine mechanisms, but possibly rather by unknown environmental factors influencing BMI in the family.

## Supplementary Data

Supplementary data are available at *IJE* online.

## Supplementary Material

Supplementary Data

## References

[1] Stene LC, Gale EA. The prenatal environment and type 1 diabetes. Diabetologia 2013;56:1888–97. 23657800 10.1007/s00125-013-2929-6

[2] Rewers M, Ludvigsson J. Environmental risk factors for type 1 diabetes. Lancet 2016;387:2340–48. 27302273 10.1016/S0140-6736(16)30507-4 PMC5571740

[3] Cardwell CR, Stene LC, Joner G Birthweight and the risk of childhood-onset type 1 diabetes: a meta-analysis of observational studies using individual patient data. Diabetologia 2010;53:641–51. 20063147 10.1007/s00125-009-1648-5

[4] Stene LC, Magnus P, Lie RT, Sovik O, Joner G, Norwegian childhood Diabetes Study G. Birth weight and childhood onset type 1 diabetes: population based cohort study. BMJ 2001;322:889–92. 11302899 10.1136/bmj.322.7291.889 PMC30582

[5] EURODIAB Substudy 2 Study Group. Rapid early growth is associated with increased risk of childhood type 1 diabetes in various European populations. Diabetes Care 2002;25:1755–60. 12351473 10.2337/diacare.25.10.1755

[6] Baum JD, Ounsted M, Smith MA. Letter: Weight gain in infancy and subsequent development of diabetes mellitus in childhood. Lancet 1975;2:866. 10.1016/s0140-6736(75)90250-0 53343

[7] Hypponen E, Kenward MG, Virtanen SM Infant feeding, early weight gain, and risk of type 1 diabetes. Childhood Diabetes in Finland (DiMe) Study Group. Diabetes Care 1999;22:1961–65. 10587826 10.2337/diacare.22.12.1961

[8] Magnus MC, Olsen SF, Granstrom C Infant growth and risk of childhood-onset type 1 diabetes in children from 2 Scandinavian birth cohorts. JAMA Pediatr 2015;169:e153759. 26642117 10.1001/jamapediatrics.2015.3759

[9] Catalano PM, Shankar K. Obesity and pregnancy: mechanisms of short term and long term adverse consequences for mother and child. BMJ 2017;356:j1. 28179267 10.1136/bmj.j1 PMC6888512

[10] Arkkola T, Kautiainen S, Takkinen HM Relationship of maternal weight status and weight gain rate during pregnancy to the development of advanced beta cell autoimmunity in the offspring: a prospective birth cohort study. Pediatr Diabetes 2011;12:478–84. 21129139 10.1111/j.1399-5448.2010.00703.x

[11] Robertson L, Harrild K. Maternal and neonatal risk factors for childhood type 1 diabetes: a matched case-control study. BMC Public Health 2010;10:281. 20507546 10.1186/1471-2458-10-281 PMC2885337

[12] Jones ME, Swerdlow AJ, Gill LE, Goldacre MJ. Pre-natal and early life risk factors for childhood onset diabetes mellitus: a record linkage study. Int J Epidemiol 1998;27:444–49. 9698133 10.1093/ije/27.3.444

[13] D’Angeli MA, Merzon E, Valbuena LF, Tirschwell D, Paris CA, Mueller BA. Environmental factors associated with childhood-onset type 1 diabetes mellitus: an exploration of the hygiene and overload hypotheses. Arch Pediatr Adolesc Med 2010;164:732–38. 20679164 10.1001/archpediatrics.2010.115 PMC3064074

[14] Hussen HI, Persson M, Moradi T. Maternal overweight and obesity are associated with increased risk of type 1 diabetes in offspring of parents without diabetes regardless of ethnicity. Diabetologia 2015;58:1464–73. 25940642 10.1007/s00125-015-3580-1

[15] Rasmussen T, Stene LC, Samuelsen SO Maternal BMI before pregnancy, maternal weight gain during pregnancy, and risk of persistent positivity for multiple diabetes-associated autoantibodies in children with the high-risk HLA genotype: the MIDIA study. Diabetes Care 2009;32:1904–06. 19592628 10.2337/dc09-0663 PMC2752934

[16] Lindell N, Carlsson A, Josefsson A, Samuelsson U. Maternal obesity as a risk factor for early childhood type 1 diabetes: a nationwide, prospective, population-based case-control study. Diabetologia 2018;61:130–37. 29098322 10.1007/s00125-017-4481-2 PMC6448943

[17] Sipetic S, Vlajinac H, Kocev N, Saji S. The Belgrade childhood diabetes study: prenatal and social associations for type 1 diabetes. Paediatr Perinat Epidemiol 2004;18:33–39. 14738545 10.1111/j.1365-3016.2004.00533.x

[18] McKinney PA, Parslow R, Gurney K, Law G, Bodansky HJ, Williams DR. Antenatal risk factors for childhood diabetes mellitus; a case-control study of medical record data in Yorkshire, UK. Diabetologia 1997;40:933–39. 9267988 10.1007/s001250050770

[19] Vlajinac H, Sipetic S, Marinkovic J, Bjekic M, Kocev N, Sajic S. The Belgrade childhood diabetes study—comparison of children with type 1 diabetes with their siblings. Paediatr Perinat Epidemiol 2006;20:238–43. 16629698 10.1111/j.1365-3016.2006.00713.x

[20] Richmond RC, Al-Amin A, Smith GD, Relton CL. Approaches for drawing causal inferences from epidemiological birth cohorts: a review. Early Hum Dev 2014;90:769–80. 25260961 10.1016/j.earlhumdev.2014.08.023 PMC5154380

[21] Smith GD. Assessing intrauterine influences on offspring health outcomes: can epidemiological studies yield robust findings? Basic Clin Pharmacol Toxicol 2008;102:245–56. 18226080 10.1111/j.1742-7843.2007.00191.x

[22] Magnus P, Irgens LM, Haug K, Nystad W, Skjaerven R, Stoltenberg C. Cohort profile: the Norwegian Mother and Child Cohort Study (MoBa). Int J Epidemiol 2006;35:1146–50. 16926217 10.1093/ije/dyl170

[23] Nilsen RM, Vollset SE, Gjessing HK Self-selection and bias in a large prospective pregnancy cohort in Norway. Paediatr Perinat Epidemiol 2009;23:597–608. 19840297 10.1111/j.1365-3016.2009.01062.x

[24] Magnus P, Birke C, Vejrup K Cohort Profile Update: The Norwegian Mother and Child Cohort Study (MoBa). Int J Epidemiol 2016;45:382–88. 27063603 10.1093/ije/dyw029

[25] Nohr EA, Frydenberg M, Henriksen TB, Olsen J. Does low participation in cohort studies induce bias? Epidemiology 2006;17:413–18. 16755269 10.1097/01.ede.0000220549.14177.60

[26] Olsen J, Melbye M, Olsen SF The Danish National Birth Cohort—its background, structure and aim. Scand J Public Health 2001;29:300–07. 11775787 10.1177/14034948010290040201

[27] Skrivarhaug T, Stene LC, Drivvoll AK, Strom H, Joner G. Incidence of type 1 diabetes in Norway among children aged 0–14 years between 1989 and 2012: has the incidence stopped rising? Results from the Norwegian Childhood Diabetes Registry. Diabetologia 2014;57:57–62. 24149838 10.1007/s00125-013-3090-y

[28] Svensson J, Lyngaae-Jorgensen A, Carstensen B, Simonsen LB, Mortensen HB. Long-term trends in the incidence of type 1 diabetes in Denmark: the seasonal variation changes over time. Pediatr Diabetes 2009;10:248–54. 19067889 10.1111/j.1399-5448.2008.00483.x

[29] Carstensen B, Kristensen JK, Marcussen MM, Borch-Johnsen K. The National Diabetes Register. Scand J Public Health 2011;39:58–61. 10.1177/1403494811404278 21775353

[30] Verbeeten KC, Elks CE, Daneman D, Ong KK. Association between childhood obesity and subsequent Type 1 diabetes: a systematic review and meta-analysis. Diabet Med 2011;28:10–18. 21166841 10.1111/j.1464-5491.2010.03160.x

[31] Ong KK. Early determinants of obesity. Endocr Dev 2010;19:53–61. 20551668 10.1159/000316897

[32] Knip M, Virtanen SM, Akerblom HK. Infant feeding and the risk of type 1 diabetes. Am J Clin Nutr 2010;91:1506S–13S. 20335552 10.3945/ajcn.2010.28701C PMC6443298

[33] Virtanen SM. Dietary factors in the development of type 1 diabetes. Pediatr Diabetes 2016;17(Suppl 22):49–55. 27411437 10.1111/pedi.12341

[34] Fourlanos S, Elkassaby S, Varney MD, Colman PG, Harrison LC. Higher body mass index in adults at diagnosis of the slowly progressive form of type 1 diabetes mellitus is associated with lower risk HLA genes. Diabetes Res Clin Pract 2014;104:e69–71. 24698405 10.1016/j.diabres.2014.03.009

[35] Yang J, Lernmark A, Uusitalo UM Prevalence of obesity was related to HLA-DQ in 2–4-year-old children at genetic risk for type 1 diabetes. Int J Obes (Lond) 2014;38:1491–96. 24694666 10.1038/ijo.2014.55 PMC4185013

[36] Field SF, Howson JM, Walker NM, Dunger DB, Todd JA. Analysis of the obesity gene FTO in 14,803 type 1 diabetes cases and controls. Diabetologia 2007;50:2218–20. 17657473 10.1007/s00125-007-0767-0 PMC2151140

[37] Bohlin J, Andreassen BK, Joubert BR Effect of maternal gestational weight gain on offspring DNA methylation: a follow-up to the ALSPAC cohort study. BMC Res Notes 2015;8:321. 26219460 10.1186/s13104-015-1286-6 PMC4518864

[38] Liu X, Chen Q, Tsai HJ Maternal preconception body mass index and offspring cord blood DNA methylation: exploration of early life origins of disease. Environ Mol Mutagen 2014;55:223–30. 24243566 10.1002/em.21827 PMC4547934

[39] Morales E, Groom A, Lawlor DA, Relton CL. DNA methylation signatures in cord blood associated with maternal gestational weight gain: results from the ALSPAC cohort. BMC Res Notes 2014;7:278. 24886386 10.1186/1756-0500-7-278 PMC4108052

[40] Pendeloski KPT, Ono E, Torloni MR, Mattar R, Daher S. Maternal obesity and inflammatory mediators: a controversial association. Am J Reprod Immunol 2017; doi: 10.1111/aji.12674. 10.1111/aji.12674 28328066

[41] Wilson RM, Messaoudi I. The impact of maternal obesity during pregnancy on offspring immunity. Mol Cell Endocrinol 2015;418 Pt 2:134–42. 26232506 10.1016/j.mce.2015.07.028 PMC4674375

[42] Munafo MR, Tilling K, Taylor AE, Evans DM, Davey Smith G. Collider scope: when selection bias can substantially influence observed associations. Int J Epidemiol 2018;47:226–35. 29040562 10.1093/ije/dyx206 PMC5837306

[43] Garfield CF, Duncan G, Gutina A Longitudinal study of body mass index in young males and the transition to fatherhood. Am J Mens Health 2016;10:Np158-np67. 26198724 10.1177/1557988315596224 PMC5293174

