# Supplementary material for: Paternal and maternal obesity but not gestational weight gain is associated with type 1 diabetes
Source: Int J Epidemiol. 2018 Feb 5;47(2):417–26. doi: 10.1093/ije/dyx266 (PMC5913633; doi:10.1093/ije/dyx266)
Supplement: Supplementary Data [file dyx266_20171120_online-only_supplement.docx]

**Supplementary Table 1. Distribution of characteristics among included and excluded eligible participants for each cohort**

| **Characteristic** | **MoBa** | | **DNBC** | |
| --- | --- | --- | --- | --- |
|  | **Excluded**  **(n= 26,859)** | **Included**  **(n=81,630)** | **Excluded**  **(n=41,951)** | **Included**  **(n= 50,701)** |
| **Maternal age (Mean(SD))** | 29.7 (5.1) | 30.2 (4.5) | 30.1 (4.4) | 30.6 (4.2) |
| **Maternal parity (n(%))** |  |  |  |  |
| Primiparous | 10,208 (38.0) | 37,413 (45.8) | 17,664 (42.1) | 23,061 (45.5) |
| 1 | 10,295 (38.3) | 28,731 (35.2) | 13,065 (31.1) | 19,004 (37.5) |
| 2+ | 6,356 (23.7) | 15,486 (19.0) | 5,329 (12.7) | 8,621 (17.0) |
| Missing | 0 (0) | 0 (0.0) | 5,893 (14.1) | 15 (0.03) |
| **Maternal education (n(%))** |  |  |  |  |
| Less than high school (MoBa)/9^th^ grade with exam (DNBC) | 2,388 (14.5) | 5,579 (6.9) | 1,133 (2.7) | 3,672 (7.2) |
| High school (MoBa) /10^th^ grade with exam (DNBC) | 5,474 (33.2) | 23,452 (28.9) | 4,109 (9.8) | 13,687 (27.0) |
| Up to 4 years of college (MoBa) / Technical school (DNBC) | 5,562 (33.7) | 33,680 (41.4) | 2,138 (5.1) | 6,886 (13.6) |
| More than 4 years of college(MoBa)/High school or more(DNBC) | 3,082 (18.7) | 18,576 (22.9) | 8,378 (20.0) | 25,969 (51.2) |
| Missing | 10,353 | 343 | 26,193 (62.4) | 507 (1.0) |
| **Maternal smoking during pregnancy (n(%))** |  |  |  |  |
| No | 11,724 (69.5) | 62,963 (77.4) | 28,668 (68.3) | 37,954 (74.9) |
| Yes | 5,158 (30.6) | 18,346 (22.6) | 11,021 (26.3) | 12,746 (25.1) |
| Missing | 9,977 | 321 | 2,262 (5.4) | 1 (0.0) |
| **Maternal diabetes (n(%))^*^** |  |  |  |  |
| No | 26,715 (99.5) | 81,268 (99.6) | 40,857 (97.4) | 49,316 (97.3) |
| Yes | 144 (0.5) | 362 (0.4) | 1,094 (2.6) | 1,385 (2.7) |
| Missing |  | 0 | 0 | 0 |
| **Paternal age (n(%))** |  |  |  |  |
| Less than 25 | 2,145 (8.0) | 3,566 (4.4) | 2,245 (5.4) | 1,656 (3.3) |
| 25-29 | 6,132 (23.0) | 18,446 (22.7) | 11,156 (26.6) | 13,184 (26.0) |
| 30-34 | 9,449 (35.4) | 31,934 (39.2) | 16,025 (38.2) | 20,011 (39.5) |
| 35+ | 8,998 (33.7) | 27,492 (33.8) | 11,663 (27.8) | 15,232 (30.0) |
| Missing | 135 | 192 | 862 (2.0) | 618 (1.2) |
| **Paternal education (n(%))** |  |  |  |  |
| Less than high school (MoBa) / 9^th^ grade with exam (DNBC) | 2,230 (14.1) | 8,026 (10.1) | 2,778 (6.6) | 9,430 (18.6) |
| High school (MoBa) / 10^th^ grade with exam (DNBC) | 6,636 (42.1) | 31,845 (40.2) | 5,188 (12.4) | 17,608 (34.7) |
| Up to 4 years of college (MoBa) / Technical school (DNBC) | 3,638 ( 23.1) | 21,728 (27.4) | 910 (2.2) | 2,784 (5.5) |
| More than 4 years of college (MoBa)/ High school or more (DNBC) | 3,262 (20.7) | 17,609 (22.2) | 6,321 (15.1) | 18,928 (37.3) |
| Missing | 11,093 | 2,422 | 26,754 (63.8) | 1,951 (3.8) |
| **Paternal smoking (MoBa) / Partner smoking (DNBC) (n(%))** |  |  |  |  |
| No | 11,785 (69.7) | 59,930 (73.8) | 24,642 (58.8) | 35,827 (70.7) |
| Yes | 5,134 (30.3) | 21,301 (26.2) | 11,402 (27.2) | 14,834 (29.3) |
| Missing | 9,940 | 399 | 5,907 (14.1) | 40 (0.1) |
| **Paternal type 1 diabetes (n(%))** |  |  |  |  |
| No | 26,717 (99.5) | 81,106 (99.4) | NA | NA |
| Yes | 142 (0.5) | 524 (0.6) | NA | NA |
| **Child gender (n(%))** |  |  |  |  |
| Male | 13,844 (51.5) | 41,796 (51.2) | 21,715 (51.8) | 25,773 (50.8) |
| Female | 13,015 (48.5) | 39,834 (48.8) | 20,236 (48.2) | 24,928 (49.2) |
| **Child birthweight (Mean(SD))** | 3.6 (0.6) | 3.6 (0.5) | 3.6 (0.6) | 3.6 (0.5) |
| Missing | 120 (0.4) | 99 (0.1) | 471 (1.1) | 251 (0.5) |

Abbreviations: BMI, body mass index; MoBa, The Norwegian Mother and Child Cohort Study; DNBC, The Danish National Birth Cohort; SD, Standard deviation.

*Maternal diabetes includes only type 1 diabetes in MoBa while it includes all forms of diabetes in DNBC.

Unless otherwise specified, data are n(%), where % is calculated among those with valid data for each variable.

**Supplementary Table 2. Associations of maternal pre-pregnancy body-mass index and paternal body-mass index with the risk of type 1 diabetes: results from the complete case analysis**

| **Parent** | **Study** | **Exposure** | **Person years** | **Events** | **Unadjusted**  **HR (95% CI)** | **Adjusted**  **HR (95% CI)** | **Test of heterogeneity (p-value)** |
| --- | --- | --- | --- | --- | --- | --- | --- |
| Mother | MoBa | Continuous | 874,066 | 286 | 1.05 (1.03, 1.08) | 1.05 (1.02, 1.07) |  |
|  |  | Underweight | 25,792 | 9 | 1.30 (0.66, 2.55) | 1.18 (0.58, 2.42) |  |
|  |  | Normal weight | 574,074 | 154 | 1 | 1 |  |
|  |  | Overweight | 192,791 | 86 | 1.65 (1.26, 2.17) | 1.57 (1.19, 2.06) |  |
|  |  | Obese | 81,408 | 37 | 1.68 (1.18, 2.41) | 1.57 (1.09, 2.27) |  |
|  | DNBC | Continuous | 701,599 | 201 | 1.01 (0.97, 1.04) | 0.99 (0.96, 1.03) |  |
|  |  | Underweight | 37,218 | 11 | 1.31 (0.71, 2.42) | 1.34 (0.73, 2.47) |  |
|  |  | Normal weight | 464,794 | 136 | 1 | 1 |  |
|  |  | Overweight | 120,027 | 31 | 0.77 (0.52, 1.14) | 0.72 (0.48, 1.07) |  |
|  |  | Obese | 79,560 | 23 | 1.36 (0.88, 2.12) | 1.19 (0.74, 1.91) |  |
|  | Combined | Continuous | 1,575,665 | 487 | 1.03 (0.99, 1.07) | 1.02 (0.96, 1.08) | 0.007 |
|  |  | Underweight | 63,010 | 20 | 1.31 (0.83, 2.06) | 1.29 (0.81, 2.05) | 0.85 |
|  |  | Normal weight | 1,038,868 | 290 | 1 | 1 | NA |
|  |  | Overweight | 312,818 | 117 | 1.14 (0.54, 2.41) | 1.08 (0.50, 2.29) | 0.002 |
|  |  | Obese | 160,968 | 60 | 1.55 (1.17, 2.04) | 1.39 (1.04, 1.86) | 0.415 |
| Father | MoBa | Continuous | 855,800 | 281 | 1.06 (1.02, 1.10) | 1.05 (1.01, 1.09) |  |
|  |  | Underweight | 1,776 | 1 | 1.99 (0.28, 14.28) | 1.88 (0.26, 13.73) |  |
|  |  | Normal weight | 384,966 | 109 | 1 | 1 |  |
|  |  | Overweight | 387,243 | 130 | 1.19 (0.92, 1.54) | 1.16 (0.89, 1.51) |  |
|  |  | Obese | 81,814 | 41 | 1.78 (1.23, 2.57) | 1.61 (1.10, 2.35) |  |
|  | DNBC | Continuous | 677,150 | 195 | 1.00 (0.96, 1.05) | 1.01 (0.96, 1.05) |  |
|  |  | Underweight | 3,383 | 1 | 1.32 (0.18, 9.49) | 1.31 (0.18, 9.36) |  |
|  |  | Normal weight | 334,990 | 100 | 1 | 1 |  |
|  |  | Overweight | 284,081 | 77 | 1.02 (0.76, 1.37) | 1.00 (0.74, 1.35) |  |
|  |  | Obese | 54,696 | 17 | 1.26 (0.75, 2.10) | 1.33 (0.80, 2.23) |  |
|  | Combined | Continuous | 1,532,950 | 476 | 1.03 (0.97, 1.09) | 1.03 (0.99, 1.07) | 0.20 |
|  |  | Underweight | 5,159 | 2 | 1.62 (0.40, 6.56) | 1.57 (0.39, 6.36) | 0.80 |
|  |  | Normal weight | 719,956 | 209 | 1 | 1 | NA |
|  |  | Overweight | 671,324 | 207 | 1.11 (0.92, 1.35) | 1.09 (0.89, 1.33) | 0.47 |
|  |  | Obese | 136,510 | 58 | 1.57 (1.14, 2.18) | 1.51 (1.11, 2.04) | 0.56 |

Maternal pre-pregnancy body-mass index adjusted for maternal age, parity, education, smoking status during pregnancy and diabetes (type 1 in MoBa and all types in DNBC). The available study sample in the multivariable analysis was 694,264 person years and 199 events from DNBC, while it was 867,387 person years and 283 events from MoBa.

Paternal body-mass index adjusted for paternal age, education, smoking (paternal smoking in MoBa and maternal partner smoking in DNBC) and type 1 diabetes (MoBa only). The available study sample in the multivariable analysis was 655,061 person years and 190 events from DNBC, while it was 829,559 person years and 275 events from MoBa.

**Supplementary Table 3. Examining the degree of mediation by birth weight and infant weight gain on the association between maternal pre-pregnancy body-mass index and the risk of type 1 diabetes: results from the multiple imputation analysis**

| **Study** | **Exposure** | **Person years** | **Events** | **Unadjusted**  **HR (95% CI)** | **Model 1**  **Adjusted**  **HR (95% CI)** | **Model 2**  **Adjusted**  **HR (95% CI)** | **Test of heterogeneity**  **(p-value)** |
| --- | --- | --- | --- | --- | --- | --- | --- |
| MoBa | Continuous | 896,679 | 293 | 1.05 (1.03, 1.08) | 1.05 (1.02, 1.07) | 1.04 (1.02, 1.07) |  |
|  | Underweight | 26,400 | 9 | 1.31 (0.67, 2.56) | 1.33 (0.68, 2.59) | 1.39 (0.71, 2.74) |  |
|  | Normal weight | 588,534 | 158 | 1 | 1 | 1 |  |
|  | Overweight | 197,983 | 88 | 1.64 (1.25, 2.15) | 1.60 (1.21, 2.10) | 1.54 (1.17, 2.03) |  |
|  | Obese | 83,762 | 38 | 1.67 (1.17, 2.40) | 1.57 (1.09, 2.27) | 1.49 (1.03, 2.15) |  |
| DNBC | Continuous | 722,194 | 206 | 1.01 (0.97, 1.04) | 1.00 (0.96, 1.03) | 0.99 (0.96, 1.03) |  |
|  | Underweight | 37,816 | 11 | 1.28 (0.69, 2.37) | 1.30 (0.70, 2.40) | 1.33 (0.72, 2.47) |  |
|  | Normal weight | 472,585 | 138 | 1 | 1 | 1 |  |
|  | Overweight | 127,823 | 33 | 0.79 (0.54, 1.17) | 0.75 (0.51, 1.11) | 0.75 (0.51, 1.10) |  |
|  | Obese | 83,970 | 24 | 1.41 (0.91, 2.18) | 1.18 (0.73, 1.88) | 1.15 (0.72, 1.85) |  |
| Combined | Continuous | 1,618,873 | 499 | 1.03 (0.99, 1.07) | 1.03 (0.98, 1.08) | 1.02 (0.97, 1.07) | 0.023 |
|  | Underweight | 64,216 | 20 | 1.29 (0.82, 2.04) | 1.31 (0.84, 2.07) | 1.36 (0.86, 2.14) | 0.93 |
|  | Normal weight | 1,061,119 | 296 | 1 | 1 | 1 | NA |
|  | Overweight | 325,806 | 121 | 1.15 (0.56, 2.36) | 1.11 (0.53, 2.33) | 1.09 (0.54, 2.20) | 0.003 |
|  | Obese | 167,732 | 62 | 1.56 (1.18, 2.06) | 1.41 (1.06, 1.89) | 1.35 (1.01, 1.81) | 0.40 |

Model 1 was adjusted for maternal age, parity, education, smoking status during pregnancy, diabetes (type 1 in MoBa and all types in DNBC).

Model 2 was adjusted for all covariate in model 1 in addition to birth weight and infant weight gain the first 12 months.

**Supplementary Table 4. Association between mutually adjusted parental body-mass index with the risk of type 1 diabetes: results from the multiple imputation analysis**

| **Parent** | **Study** | **Exposure** | **Person years** | **Events** | **Unadjusted HR (95% CI)** | **Adjusted HR**  **(95% CI)** | **Test of heterogeneity**  **(p-value)** |
| --- | --- | --- | --- | --- | --- | --- | --- |
| Mother | MoBa | Underweight | 26,400 | 9 | 1.31 (0.67, 2.56) | 1.36 (0.69, 2.66) |  |
|  |  | Normal weight | 588,534 | 158 | 1 | 1 |  |
|  |  | Overweight | 197,983 | 88 | 1.64 (1.25, 2.15) | 1.53 (1.16, 2.03) |  |
|  |  | Obese | 83,762 | 38 | 1.67 (1.17, 2.40) | 1.40 (0.95, 2.06) |  |
|  | DNBC | Underweight | 37,816 | 11 | 1.28 (0.69, 2.37) | 1.30 (0.70, 2.40) |  |
|  |  | Normal weight | 472,585 | 138 | 1 | 1 |  |
|  |  | Overweight | 127,823 | 33 | 0.79 (0.54, 1.17) | 0.76 (0.51, 1.12) |  |
|  |  | Obese | 83,970 | 24 | 1.41 (0.91, 2.18) | 1.18 (0.74, 1.90) |  |
|  | Combined | Underweight | 64,216 | 20 | 1.29 (0.82, 2.04) | 1.33 (0.84, 2.09) | 0.92 |
|  |  | Normal weight | 1,061,119 | 296 | 1 | 1 | NA |
|  |  | Overweight | 325,806 | 121 | 1.15 (0.56, 2.36) | 1.09 (0.55, 2.17) | 0.004 |
|  |  | Obese | 167,732 | 62 | 1.56 (1.18, 2.06) | 1.31 (0.97, 1.76) | 0.583 |
| Father | MoBa | Underweight | 1,868 | 1 | 1.97 (0.27, 14.10) | 1.91 (0.27, 13.68) |  |
|  |  | Normal weight | 402,983 | 113 | 1 | 1 |  |
|  |  | Overweight | 405,868 | 136 | 1.19 (0.92, 1.54) | 1.13 (0.87, 1.46) |  |
|  |  | Obese | 85,961 | 43 | 1.78 (1.24, 2.57) | 1.49 (1.02, 2.20) |  |
|  | DNBC | Underweight | 3,824 | 1 | 1.22 (0.17, 8.73) | 1.19 (0.17, 8.44) |  |
|  |  | Normal weight | 351,465 | 104 | 1 | 1 |  |
|  |  | Overweight | 304,472 | 82 | 1.04 (0.77, 1.40) | 1.06 (0.79, 1.43) |  |
|  |  | Obese | 62,433 | 19 | 1.34 (0.81, 2.21) | 1.35 (0.82, 2.24) |  |
|  | Combined | Underweight | 5,692 | 2 | 1.55 (0.38, 6.25) | 1.51 (0.38, 6.01) | 0.74 |
|  |  | Normal weight | 754,448 | 217 | 1 | 1 | NA |
|  |  | Overweight | 710,340 | 218 | 1.12 (0.92, 1.37) | 1.10 (0.90, 1.34) | 0.75 |
|  |  | Obese | 148,394 | 62 | 1.61 (1.20, 2.17) | 1.44 (1.06, 1.95) | 0.76 |

Adjusted for maternal age, parity, education, smoking status during pregnancy and diabetes (type 1 in MoBa and all types in DNBC), in addition to paternal age, education, smoking (paternal smoking in MoBa and maternal partner smoking in DNBC), type 1 diabetes (MoBa only) and maternal/paternal BMI.

**Supplementary Table 5. Association between maternal total gestational weight gain with the risk of type 1 diabetes: results from the complete case analysis**

| **Study** | **Person years** | **Events** | **Mean (SD)** | **Unadjusted**  **HR (95% CI)** | **Adjusted**  **HR (95% CI)** | **Test of heterogeneity**  **(p-value)** |
| --- | --- | --- | --- | --- | --- | --- |
| MoBa | 829,122 | 266 | 14.9 (5.8) | 1.00 (0.98, 1.02) | 1.00 (0.98, 1.02) |  |
| DNBC | 716,614 | 205 | 14.9 (5.9) | 1.00 (0.97, 1.03) | 1.00 (0.98, 1.03) |  |
| Combined | 1,545,736 | 471 | 14.9 | 1.00 (0.98, 1.02) | 1.00 (0.99, 1.02) | 1.00 |

Adjusted for maternal age, parity, education, smoking status during pregnancy, diabetes (type 1 in MoBa and all types in DNBC), weight at the start of pregnancy and child gender. The available study sample in the multivariable analysis was 688,684 person years and 198 events from DNBC, while it was 822,930 person years and 263 events from MoBa.

Including a second degree term indicated no evidence of any nonlinear associations (p-value 0.36 in MoBa and 0.07 in DNBC).

**Supplementary Figure 1. Risk of type 1 diabetes among those included and excluded in the Norwegian Mother and Child Cohort Study**

The hazard ratio for type 1 diabetes was 1.03 (95 % CI: 0.81, 1.30) when comparing included to excluded eligible participants in MoBa.

**Supplementary Figure 2. Risk of type 1 diabetes among those included and excluded in the Danish National Birth Cohort**

The hazard ratio for type 1 diabetes was 1.13 (95 % CI: 0.92, 1.39) when comparing included to excluded eligible participants in DNBC.
